# Presynaptic GABA_B_ receptors reduce transmission at parabrachial synapses in the lateral central amygdala by inhibiting N-type calcium channels

**DOI:** 10.1038/srep19255

**Published:** 2016-01-12

**Authors:** A.J. Delaney, J.W. Crane

**Affiliations:** 1 School of Biomedical Sciences, Charles Sturt University, NSW, Australia

## Abstract

The nocioceptive information carried by neurons of the pontine parabrachial nucleus to neurons of the lateral division of the central amydala (CeA-L) is thought to contribute to the affective components of pain and is required for the formation of conditioned-fear memories. Importantly, excitatory transmission between parabrachial axon terminals and CeA-L neurons can be inhibited by a number of presynaptic receptors linked to Gi/o-type G-proteins, including α2-adrenoceptors and GABA_B_ receptors. While the intracellular signalling pathway responsible for α2-adrenoceptor inhibition of synaptic transmission at this synapse is known, the mechanism by which GABA_B_ receptors inhibits transmission has not been determined. The present study demonstrates that activation of presynaptic GABA_B_ receptors reduces excitatory transmission between parabrachial axon terminals and CeA-L neurons by inhibiting N-type calcium channels. While the involvement of G_βγ_ subunits in mediating the inhibitory effects of GABA_B_ receptors on N-type calcium channels is unclear, this inhibition does not involve G_βγ_-independent activation of pp60C-src tyrosine kinase. The results of this study further enhance our understanding of the modulation of the excitatory input from parabrachial axon terminals to CeA-L neurons and indicate that presynaptic GABA_B_ receptors at this synapse could be valuable therapeutic targets for the treatment of fear- and pain-related disorders.

The laterocapsular division of the central amygdala (CeA-LC) is referred to as the ‘nocioceptive amygdala’^1^. This region receives nocioceptor-related information from spinal cord lamina I neurons, via neurons of the pontine parabrachial nucleus^2^,^3^, and contains a high proportion of neurons that respond to peripherally applied noxious stimuli^4^. As the central amygdala has a well-established role in the generation and modulation of emotional responses, the processing of nocioceptive information within the CeA-LC is thought to underpin the generation of the emotional/affective components of the pain experience^1^,^2^. In support of this suggestion, disruption of the parabrachial connection to CeA-LC neurons blocks the processing of the aversive component of footshock-related nocioceptor-input required for the formation of conditioned fear memories^5^.

Parabrachial neurons send direct projections to CeA-LC neurons^3^,^6^, and the release of glutamate from parabrachial axon terminals excites CeA-LC neurons via the activation of post-synaptic AMPA receptors^7^,^8^. However, glutamate release from parabrachial axon terminals is modulated by a number of presynaptic, G-protein-linked receptors. Activation of presynaptic Group I metabotropic glutamate receptors enhances transmission through an action to increase the probability of synaptic vesicle release^8^, whereas activation of Group II or III metabotropic glutamate receptors inhibits transmission by reducing release probability^9^,^10^. Similarly, activation of presynaptic α2-adrenoceptors and GABA_B_ receptors inhibits synaptic transmission between parabrachial axons and CeA-LC neurons^7^.

Binding of noradrenaline to α2-adrenoceptors on parabrachial axon terminals activates a Gi/o class of G-protein, releasing G_α_ and G_βγ_ subunits. Inhibition of the glutamate release from parabrachial axon terminals then occurs via a direct action of G_βγ_ on release machinery (i.e. SNARE complexes) to inhibit fusion of synaptic vesicles and subsequent release of neurotransmitter (i.e. glutamate), reducing the number of released vesicles but not release probability^7^. In contrast, GABA_B_ receptor-mediated inhibition of release targets calcium influx through the voltage-gated calcium channels, resulting in a reduction in release probability^7^. However, the intra-cellular signalling pathway responsible for GABA_B_-mediated inhibition of voltage-gated calcium channels has not been determined. As such, the present study sought to elucidate the intracellular mechanism by which the activation of presynaptic GABA_B_ receptors inhibits transmission between parabrachial axons and CeA-LC neurons.

## Results

**Figure 1:**
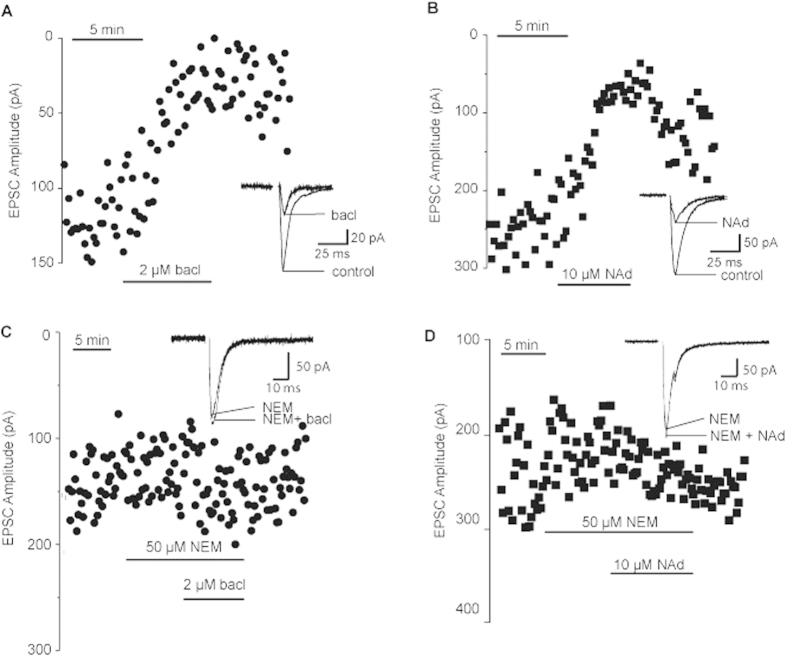
GABA_B_-receptor and noradrenenergic inhibition of parabrachial-CeA-L EPSCs is blocked by pre-incubation with 50 μM N-ethylmaleimide (NEM) for 10 minutes prior to application of baclofen or noradrenaline. (**A**) Parabrachial-CeA-L EPSC amplitudes during the application of 2 μM baclofen (bacl); inset - average of control and baclofen responses. (**B**) Parabrachial-CeA-L EPSC amplitudes during the application of 10 μM noradrenaline (NAd); inset - average of control and noradrenaline responses. (**C,D**) Parabrachial-CeA-L EPSC amplitudes in control, and with subsequent application of NEM followed by baclofen (bacl) (**C**) and noradrenaline (NAd) (**D**). Inset – average traces in NEM (NEM) and NEM + baclofen (NEM+ bacl) or noradrenaline (NEM + NAd) shown.

The axons of parabrachial neurons form perisomatic basket-synapses onto neurons of the lateral central amygdala (CeA-L)^6^. These synapses provide a significant, AMPA-mediated, excitatory input onto CeA-L neurons that is inhibited by the activation of pre-synaptic α2-adrenoceptors (by noradrenaline) and GABA_B_-receptors (by baclofen) (Fig. 1A,B; see also^7^). To determine the G proteins coupled to GABA_B_-receptors and α2-adrenoceptors located on parabrachial axon terminals, slice were pre-exposed to the sulfhydryl alkylating agent *N*-ethylmaleimide (NEM) at a concentration that selectively inhibits signalling by pertussis-toxin sensitive Gi/o-type G proteins (50 μM, 10 min bath application)^11^. The pre-incubation of slices with NEM blocked the inhibitory effects of both baclofen and noradrenaline at these synapses (Fig. 1C,D, n = 5 and 4 respectively), a result consistent with the coupling of GABA_B_-receptors and α2-adrenoceptors to Gi/o-type G proteins.

Given that both pre-synaptic GABA_B_-receptors and α2-adrenoceptors signal through Gi/o proteins, we tested whether intracellular signalling pathways were shared between these two types of receptors. The specific GABA_B_-receptor antagonists CGP5535A (3 μM) completely blocked the inhibitory effect of baclofen (2 μM; n = 3 not shown), but not the inhibitory effect of noradrenaline on synaptic transmission between parabrachial axon terminals and CeA-L neurons (10 μM, 63.9 ± 10.3% inhibition in CGP5535A compared to 70.9 ± 3.8% in control, n = 4 and 21 respectively, p < 0.05 for both). Similarly, the α2-adrenoceptor antagonist yohimbine blocked the inhibitory effect of noradrenaline, but not the inhibitory effects of baclofen (n = 5, not shown), on transmission at this synapse (average 79.6 ± 6.0% inhibition in yohimbine compared to 75.8 ± 4.1% in control, n = 3 and 7 respectively). In addition, synaptic transmission was further inhibited by both the application of noradrenaline in the presence of baclofen (44.5 ± 7.0% inhibition, n = 5, data not shown) and the application of baclofen in the presence of noradrenaline (62.6 ± 7.0% inhibition, p < 0.05, n = 5, not shown). These results indicate that while GABA_B_-receptors and α2-adrenoceptors share a common class of G-proteins (Gi/o) they mediate their effects on vesicular release from parabrachial axon terminals via distinct signal transduction mechanisms.

**Figure 2:**
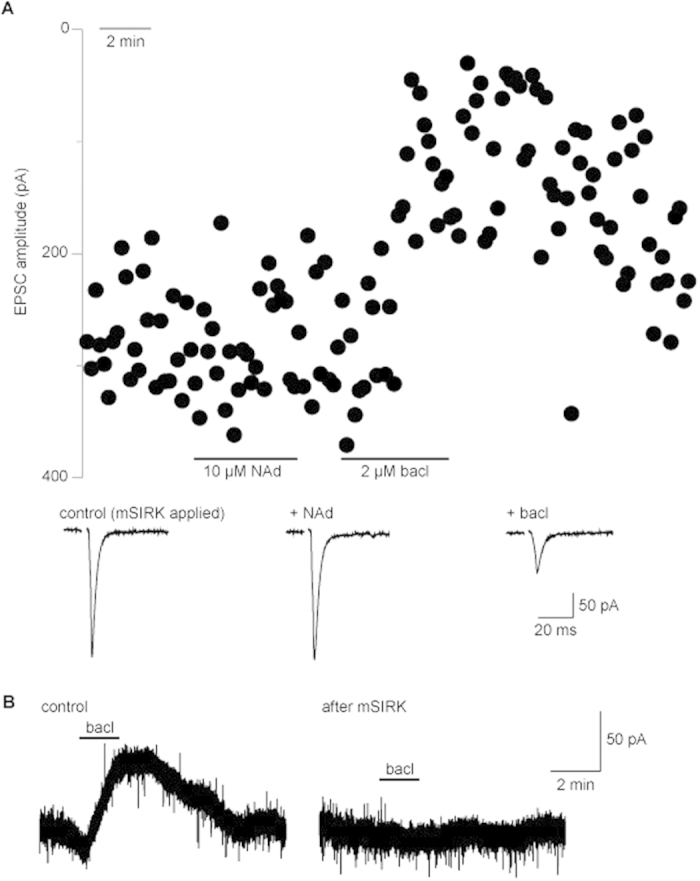
GABA_B_-mediated inhibition does not require G_βγ_ signalling. (**A**) Parabrachial-CeA-L EPSC amplitude recorded from synapses loaded with the myristlated compound mSIRK in control conditions and after the addition of noradrenaline (NAd) and baclofen (bacl). Below - average EPSCs in each condition. (**B**) Baclofen (bacl) activated post-synaptic currents recorded from CeA-L cells in control conditions and after mSIRK application.

The application of the myristilated G_βγ_ binding peptide mSIRK to terminals blocks the inhibitory effect of noradrenaline on vesicular release from parabrachial axons (Fig. 2A; see also^7^), indicating that G_βγ_ mediates the α2-adrenoceptor modulation of synaptic transmission between parabrachial axons and CeA-L neurons. In contrast, application of mSRIK failed to block the inhibitory effect of baclofen on synaptic transmission at this synapse (Fig. 2A; 71.1 ± 4.7% inhibition, p < 0.05, n = 4). However, mSIRK did block a baclofen-induced potassium current in neurons of the basolateral amygdala, demonstrating the G_βγ_ does mediate some of the effects produced by GABA_B_-receptor activation (Fig. 2B; n = 3). Therefore, while GABA_B_ receptors can use G_βγ_ to influence downstream targets, the results of this experiment suggest that G_βγ_ might not be required for GABA_B_-mediated inhibition of synaptic transmission between parabrachial axons and CeA-L neurons.

**Figure 3:**
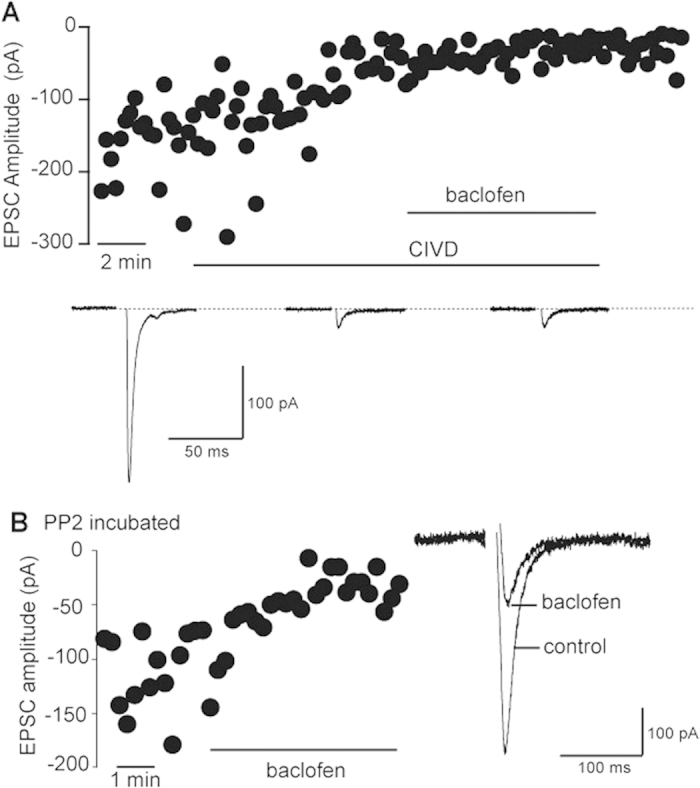
GABA_B_-mediated inhibition of parabrachial-CeA-L EPSCs by baclofen is reduced by blocking N-type calcium channels but not by pre-incubation with the selective src-kinase inhibitor PP2. (**A**) Parabrachial-CeA-L EPSC amplitudes recorded from synapses in control conditions and following sequential addition of ω-conotoxin CVID (CIVD; 200 nM) and baclofen (bacl; 2 μM). (**B**) Parabrachial-CeA-L EPSC amplitudes recorded from synapses in slices pre-incubated for 90 min in PP2 in the absence and presence of baclofen. Average EPSCs in control and baclofen are shown on right.

Baclofen reduces transmitter release from parabrachial axons by reducing voltage-dependent calcium influx into terminals resulting in a decrease in the probability of action-potential-driven vesicle fusion and neurotransmitter release^7^, and the previous experiments indicated that this GABA_B_-mediated inhibition might not involve G_βγ_ signalling. Indeed, activation of GABA_B_ receptors can produce a voltage-independent block of the N-type calcium channels via a G_βγ_-independent activation of the pp60C-src tyrosine kinase^12^. To determine whether a similar intracellular signalling process was responsible for the GABA_B_-mediated inhibition of vesicle release from parabrachial axon terminals the specific N-type calcium channel antagonist ω-conotoxin CVID^13^ (200 nM) was applied to slices. This revealed that N-type calcium channels were largely responsible for mediating neurotransmitter release from the parabrachial axon terminals (69.6 ± 8.0% inhibition; Fig. 3A, n = 5, p < 0.5). In addition, application baclofen in the presence ω-conotoxin CIVD did not produce any further inhibition of synaptic transmission (Fig. 3A, 9.2 ± 22.3% change, n = 4). Thus, activation of presynaptic GABA_B_ receptor on parabrachial axon terminals inhibits neurotransmitter release by inactivating N-type calcium channels. However, pre-incubation of the slices in a src-kinase antagonist (PP2; 10μM) did not block GABA_B_-mediated inhibition of transmission between parabrachial axons and CeA-L neurons (63.5 ± 6.2% inhibition p < 0.01, n = 8; Fig. 3B). Therefore, inhibition of neurotransmitter release from parabrachial axon terminals does not involve a voltage-independent block of the N-type calcium channels via a G_βγ_-independent activation of the pp60C-src tyrosine kinase.

## Discussion

Activation of presynaptic GABA_B_ receptors inhibits excitatory transmission between parabrachial axon terminals and CeA-L neurons^7^. The results of the present study indicate that this inhibition involves the activation of Gi/o-linked G-proteins resulting in the inactivation of N-type calcium channels responsible for action-potential-driven neurotransmitter release. Further, the inactivation of N-type calcium channels within parabrachial axon terminals is not due to the activation of pp60C-src tyrosine kinase.

This study adds to the list of Gi/o-linked receptors that act presynaptically to inhibit synaptic transmission between parabrachial axons and CeA-L neurons. Presynaptic α2-adrenoceptors, GABA_B_ receptors, and Group II and Group III metabotropic glutamate receptors all inhibit the release of synaptic vesicles from axon terminals of parabrachial neurons within the CeA-LC. While these four receptors types link to Gi/o-linked G-proteins, they inhibit synaptic transmission in different ways. Binding noradrenaline to α2-adrenoceptors on parabrachial axon terminals activates Gi/o-linked G-proteins, releasing G_α_ and G_βγ_ subunits. Inhibition of the glutamate release from axon terminals then occurs via a direct action of G_βγ_ subunits on release machinery (i.e. SNARE complexes) to inhibit fusion of synaptic vesicles and subsequent release of neurotransmitter (i.e. glutamate). This mechanism reduces the number of vesicles released from the terminal without reducing release probability^7^. In contrast, activation of GABA_B_ receptors, Group II or Group III metabotropic glutamate receptors reduces the probability of synaptic vesicle release from parabrachial axon terminals. While the mechanism by which metabotropic glutamate receptors influence release probability of parabrachial axon terminals remains to be determined, the present study demonstrates that GABA_B_ receptors reduce release probability at these terminals by inhibiting N-type calcium channels.

In the majority of instances, the activation of Gi/o-linked G-proteins inhibits the opening of presynaptic N-type calcium channels in a voltage-dependent manner^14^. This voltage-dependent inhibition involves the binding of G_βγ_ subunits to α1-domain of N-type calcium channels to stabilise the closed state of the channel, making it ‘reluctant’ to open^15^,^16^,^17^. In contrast, the present study found that application of the myristilated G_βγ_ binding peptide mSIRK did not prevent the GABA_B_-mediated reduction in synaptic transmission between parabrachial terminals and CeA-L neurons. This result suggests that G_βγ_ subunits are not involved in the inhibition of N-type calcium channels in parabrachial axon terminals. That mSIRK was able to prevent α2-adrenoceptor-mediated inhibition of synaptic transmission at this synapse demonstrates the ability of mSIRK to infiltrate parabrachial terminals within the CeA-L. However, GABA_B_-receptors form a complex with G-proteins and N-type calcium channels^18^, and the vesicular release protein syntaxin 1A form a complex with both G_βγ_ subunit and the α1-domain of N-type calcium channels^19^. These two complexes facilitate the interaction between GABA_B_-receptors, G_βγ_ subunits, and N-type calcium channels, and quite possibly limit the ability of mSIRK to bind to G_βγ_ subunits before they bind to and inhibit N-type calcium channels. Therefore, involvement of G_βγ_ subunits in GABA_B_-mediated inhibition of synaptic transmission between parabrachial terminals and CeA-L neurons warrants further investigation.

The present study did not find any evidence for a voltage-independent block of the N-type calcium channels via a G_βγ_-independent activation of the pp60C-src tyrosine kinase^12^. In addition, the results presented here demonstrate that GABA_B_-mediated inhibition of N-type calcium channels is not due to G-protein-triggered activation of a number of other kinases that are also blocked by PP2 (e.g. Lck, CSK, Eph-A2, FGF-R1, p38α MAPK, CK1δ and RIP2)^20^.

Nocioceptive information carried by neurons of the parabrachial nucleus to neurons of the CeA-LC is critical for the formation of conditioned fear memories and is thought to play a key role in generating the affective components of the pain experience^2^,^5^. Consistent with this, pharmacological interventions that alter synaptic transmission between parabrachial axon terminals and CeA-LC neurons influence nocioceptive processing and fear-memory formation. For instance, activation of α2-adrenoceptors within the central amygdala appears to decrease the affective component of visceral pain^21^, while blockade of α2-adrenoceptors within the central amygdala attenuates stress induced analgesia^22^. Further, the blockade of N-type calcium channels within the central amygdala inhibits the formation of conditioned-fear memories^23^. The results of the present study reveal that GABA_B_-receptors reduce synaptic transmission between parabrachial axon terminals and CeA-L neurons by inhibiting N-type calcium channels. Therefore, it is likely that activation of GABA_B_-receptors within the central amygdala will modulate both fear-memory formation and pain perception, making presynaptic GABA_B_-receptors within the CeA-L valuable therapeutic targets for the treatment of fear-and pain-related disorders.

## Methods

Coronal brain sections were prepared from 21–28 day old Wistar rats. Rats were anaesthetized using isoflurane, decapitated, and their brains removed into ice cold artificial cerebrospinal fluid (ACSF) solution containing (in mM): 118 NaCl, 25 NaHCO_3_, 10 Glucose, 2.5 CaCl_2_, 1.2 NaH_2_PO_4_ and 1.3 MgCl_2_. Brains were sliced into 350 μm thick coronal sections using a Leica VT1000S vibratome at 0 °C, transferred to a chamber containing ACSF at 33–34 °C for 30 mins, then maintained for several hours in ACSF at room temperature. All procedures were performed with the approval of the Institutional Animal Ethics Committees of The University of Queensland and Charles Sturt University, and carried out in accordance with the approved guidelines.

Whole cell recordings were made from brain slices maintained at 32-33 °C in a recording chamber continuously perfused with oxygenated ACSF and visualized using IR/DIC techniques. Recording electrodes (3-5 MΩ) were filled with pipette solution containing (mM): CsMeSO_4_ 135, NaCl 8, HEPES 10, Mg_2_ATP 2 and Na_3_GTP 0.3 (pH 7.2 with CsOH, osmolarity 290 mOsm/kg). Whole cell voltage-clamp recordings were made using a patch clamp amplifier (Multiclamp 700A, Axon instruments, Foster City, CA). Current signals were filtered at 4-8 kHz and digitized at 20 kHz (National Instruments, USB-6221 digitiser), acquired, stored and analyzed on Toshiba Satellite Pro L70 PC using Axograph software. Access resistance (5-15 MΩ) was monitored throughout the experiment. In all experiments, fast GABAergic transmission was blocked with picrotoxin (100 μM) added to the external ACSF solution.

Most drugs were added to the ACSF solution used to perfuse the slide i.e. picrotoxin (Sigma); CGP55845A, *N*-ethylmaleimide (NEM), yohimbine, prazosin, and NBQX (all from (Tocris); and conotoxin CIVD (a gift from Prof David Adams). myristilated G_βγ_ binding peptide, mSIRK, (Tocris) was applied directly to the parabrachial terminals by puffer pipette for 10–15 min prior to recording. The src-kinase-specific antagonist PP2 (Tocris) was applied, in ACSF, to the slices for 30–90 min in a small incubation chamber before being removed to the recording chamber.

Excitatory post-synaptic currents (EPSCs) were evoked using a bipolar stimulating electrode placed onto the surface of the slice. Responses shown are averages of 10–50 individual trials. Student’s *t* tests were used for statistical comparisons between groups (except where indicated). All results are expressed as mean ± s.e.m.

## Additional Information

**How to cite this article**: Delaney, A.J. and Crane, J.W. Presynaptic GABA_B_ receptors reduce transmission at parabrachial synapses in the lateral central amygdala by inhibiting N-type calcium channels. *Sci. Rep.* **6**, 19255; doi: 10.1038/srep19255 (2016).
